# Efficient and Facile Synthetic Route of MoO_3_:MoS_2_ Hybrid Thin Layer via Oxidative Reaction of MoS_2_ Nanoflakes

**DOI:** 10.3390/nano12183171

**Published:** 2022-09-13

**Authors:** Hind Lamkaouane, Hajar Ftouhi, Mireille Richard-Plouet, Nicolas Gautier, Nicolas Stephant, Mimoun Zazoui, Mohammed Addou, Linda Cattin, Jean Christian Bernède, Yamina Mir, Guy Louarn

**Affiliations:** 1 Laboratoire Matériaux, Energie et Contrôle Système, Faculté des Sciences et Techniques Mohammedia, Université Hassan II de Casablanca, BP 146, Mohammedia 28806, Morocco; 2 Institut des Matériaux de Nantes Jean Rouxel (IMN), Centre National de la Recherche Scientifique (CNRS), Nantes Université, CEDEX 03, 44000 Nantes, France; 3 Équipe de Recherche Couches Minces et Nanomatériaux, Faculté des Sciences et Techniques, Université Abdelmalek Essaâdi, BP 416, Tanger 90040, Morocco; 4 MOLTECH Anjou, Unité Mixte de Recherche (UMR 6200), Centre National de la Recherche Scientifique (CNRS), Nantes Université, 2 rue de la Houssinière, 44000 Nantes, France

**Keywords:** hybrid layer, thin films, 2D nanomaterials, transition metal dichalcogenide, molybdenum disulfide, molybdenum trioxide, partial oxidation

## Abstract

In the present study, MoO_3_:MoS_2_ hybrid thin layers have been synthesized through partial oxidation of MoS_2_. We have demonstrated that the reaction requires darkness conditions to decrease the oxidation rate, thus obtaining the hybrid, MoO_3_:MoS_2_. A simple liquid-phase exfoliation (LPE) is carried out to achieve homogenous MoS_2_ nanoflakes and high reproducibility of the results after MoS_2_ oxidation. XPS analyses reveal the presence of MoO_3_, MoS_2_, and MoOxSy in the hybrid layer. These results are also confirmed by X-ray diffraction and high-resolution TEM. Optical absorbance reveals that the absorption peaks of the MoO_3_:MoS_2_ hybrid are slightly redshifted with the appearance of absorption peaks in the near-infrared region due to the defects created after the oxidation reaction. The composition and atomic percentages of each component in the hybrid layer as a function of reaction time have also been reported to give perspective guides for improving electronic and optoelectronic devices based on 2D-MoS_2_.

## 1. Introduction

From the success of graphene to the development of transition metal dichalcogenides (TMDs), inorganic compounds with layered structures have received impressive attention because of the unique properties that meet in two-dimensional structures (2D). Among the 2D nanomaterials, TMDs consisted of MX_2_, where M is a transition metal (M: Mo, W, etc.), and X is chalcogen (X: S, Se, and Te). As one of the inorganic graphene analogs, its layer nature is characterized by a strong in-plane bonding and weak Van der Waals interaction between the layers [1,2,3]. Molybdenum disulfide (MoS_2_) is categorized as a semi-conducting material. Its stable structure consists of hexagonal layers co-bonded via Van der Waals forces, and each layer has covalent bonds between Mo and S (S-Mo-S). The tunable bandgap energy from 1.2 eV for bulk MoS_2_ material to 1.8 eV in monolayer and the transition of the bandgap from indirect to direct bandgap has generated massive attention in exploring MoS_2_ for various applications, such as hydrogen production [4,5,6], optoelectronic [7,8], lubrication [9], batteries [10], photocatalysis [11], and transistors [12].

It is worth noting that over the past years, many efforts have been devoted to exploring MoS_2_ as an absorber layer for photovoltaic cells (PVs) since MoS_2_ can efficiently absorb visible light. However, the failure of this approach brings the scientists to develop another way to use MoS_2_ as a hole transport layer (HTL) in organic photovoltaic cells (OPVs) [8]. Currently, MoS_2_ represents an alternative material that could combine several essential characteristics of an ideal interlayer for the next solar cell generation and OLED applications [13,14,15,16]. Thanks to the development achieved in terms of a synthetic route to obtain controllable synthesis and a large-scale and uniform atomic layer. Among the preparation techniques of the MoS_2_ layer, we can find liquid-phase exfoliation (LPE) [17], sol-gel/spin-coating [18], thermal decomposition of ammonium tetrathiomolybdate ((NH_4_)_2_MoS_4_)/spin-coating [19], electrochemical process [8], and chemical vapor deposition (CVD) [20]. However, the use of a pure MoS_2_ thin layer is still unsatisfactory to reach a good band matching between the electrode and active layer in OPVs and a high electrochemical performance and electrode stability for LIBs. Thus, it opened up the use of heteroatomic doping or combination of 2D-MoS_2_ with another material, such as MoS_2_/Polyaniline and MoS_2_/C for LIBs [21], MoS_2_/WS_2_ for photoelectrochemical water oxidation [5], and MoS_2_/PEDOT:PSS and MoO_3_:MoS_2_ as HTL for OPVs [22,23].

The interesting properties of n-type semiconductor molybdenum oxide (MoO_3_) offer a highly effective combination with the MoS_2_ layer, and this hybrid can be helpful and useful for many applications. For this purpose, Yun et al. reported an oxidation/exfoliation process to prepare the MoO_3_:MoS_2_ hybrid as HTL for polymer solar cells; this oxidation/exfoliation technique can replace the disadvantage of classical methods of MoO_3_ thin-layer preparation, such as sol-gel and spray pyrolysis. The solution processable MoO_3_:MoS_2_ hybrid offers a high OPV performance compared with only the MoO_3_ thin layer or the MoS_2_ thin layer as HTL [23]. Another way to elaborate the MoO_3_:MoS_2_ hybrid layer is the use of electrochemical deposition method; this work was done in our laboratory, and the hybrid layer was applied in planar OPVs as HTL. The hybrid synthesized combines both advantages of the use of MoO_3_ as an efficient hole transporter layer and MoS_2_ as a good electron extractor layer [8]. D. Lei et al. reported a synthesis by the hydrothermal method of MoS_2_/ carbon shells and oxidation of the MoS_2_ which gives a composite consisting of hybrid MoS_2_-MoO_x_ combined with carbon shells for lithium-sulfur batteries. The use of MoS_2_-MoO_x_ results in the enhancement of Li-S battery performances because first, the MoS_2_ coupled with MoO_x_ can improve the absorption toward polysulfides. Additionally, the heterostructure can provide better electron mobility and high catalytic activity, which can promote the redox reaction of polysulfide [10]. Inspired by these previous works, we report a facile and controllable synthetic strategy to prepare the hybrid MoO_3_:MoS_2_ layer. The synthesis is achieved by exfoliation/oxidation of MoS_2_ using H_2_O_2_ as an oxidizer. Hydrogen peroxide (H_2_O_2_) promotes a facile, fast, and low-temperature MoS_2_ oxidation, which is why it is often employed as an oxidizing agent for 2D materials, including MoS_2_ [23]. Based on our previous work, the MoO_3_:MoS_2_ hybrid thin film as HTL synthesized by the chemical vapor deposition coupled rapid thermal annealing (CVD-RTA) technique reaches good PV performance when the MoO_3_ content exceeds 60%. This indicates the importance of controlling the hybrid composition. Therefore, in our work, the idea behind the use of H_2_O_2_ as an oxidizer is to discover and provide a controlled method to synthesize the MoO_3_:MoS_2_ hybrid thin layer and to reach a high content percentage of MoO_3_ in the prepared hybrid thin film [24]. The free organic solvent liquid phase exfoliation was adopted to obtain MoS_2_ nanoflakes, and a homogeneous hybrid layer was grown on a flexible substrate from aqueous dispersion via simple coating/centrifugation techniques. The percentage ratio of MoO_3_:MoS_2_ was controlled through the control of H_2_O_2_ concentration and reaction duration at ambient conditions. Moreover, the structural, morphological, and optical properties of the hybrid MoO_3_:MoS_2_ were studied as well.

## 2. Materials and Methods

### 2.1. Experimental Method

The experimental method in the present study was divided into two main parts. Before working on the exfoliated MoS_2_ as the starting reactive to produce the hybrid thin film and evaluate their structural, optical, and morphological properties, the partial oxidation was first checked with the MoS_2_ nanopowder.

#### 2.1.1. Partial Oxidation of Nanodispersed MoS_2_

The hybrid MoO_3_:MoS_2_ nanopowder was prepared through partial oxidation of molybdenum disulfide (MoS_2_) using hydrogen peroxide (H_2_O_2_) as the oxidant. MoS_2_ nano-powder (90 nm) was purchased from Sigma Aldrich (ref: 804169). In a beaker of 25 mL, MoS_2_ (5 mg) was dispersed in distilled water with molar mass of C_MoS__2_ = 6 × 10^−3^ mol L^−^^1^ under constant and continuous magnetic stirring for 48 h at 50 °C. During stirring, the beaker was covered by parafilm to avoid any contact with dust and moisture. Then, H_2_O_2_ was added to the MoS_2_ dispersion with a volume ratio of 3:1 *v/v* under stirring at ambient temperature. The reaction was performed in daylight or in the dark. In the dark condition, the beaker containing the solution was totally covered by aluminum foil. After the reaction, the solution (2 mL) was drop-casted on glass and/or ITO-coated glass substrates, followed by drying on a hotplate at 80 °C for 30 min for XPS analysis and further characterizations. The reaction times were taken as variable parameters to control the evolution of the reaction and to subsequently investigate their effect on the hybrid stoichiometry.

#### 2.1.2. Preparation of MoS_2_ and MoO_3_:MoS_2_ Hybrid Thin Films

MoS_2_ exfoliation: Using the LPE method, MoS_2_ was simply exfoliated in distilled water. The MoS_2_ nanopowder (160 mg) was stirred in distilled water with initial mass concentration of 8 mg·mL^−^^1^ for 2 h at 80 °C. Then, the dispersion was transferred to a beaker of 25 mL, placed inside a water bath, and sonicated for 6 h using an ultrasonic processor (bioblock scientific, vibra cell) with 500 W maximum power, and 20% amplitude of power with 6 s on and 3 s off pulses. Throughout the whole sonication process, the temperature was kept between 40 °C and 60 °C. The resulting green-dark suspension was settled without disturbance for 2 h, and the upper suspension was collected and centrifuged for 30 min to obtain MoS_2_ nanosheets. Two solutions were obtained depending on the centrifugation speed at 2377 rpm, which corresponds to 600× *g* relative centrifugal forces (RCF), and 3069 rpm, which corresponds to 1000× *g*. The final mass concentrations are 1 mg mL^−1^ and 0.8 mg mL^−1^ obtained using relative centrifugation force of 600× *g* and 1000× *g*, respectively, and were estimated from the final mass of the unexfoliated nonpowered. The supernatants containing nanosheets of MoS_2_ exfoliated/centrifuged at 1000× *g* were carefully collected and directly transferred to another centrifuge tube to proceed with a centrifugation-coating, described below.

**Figure 1:**
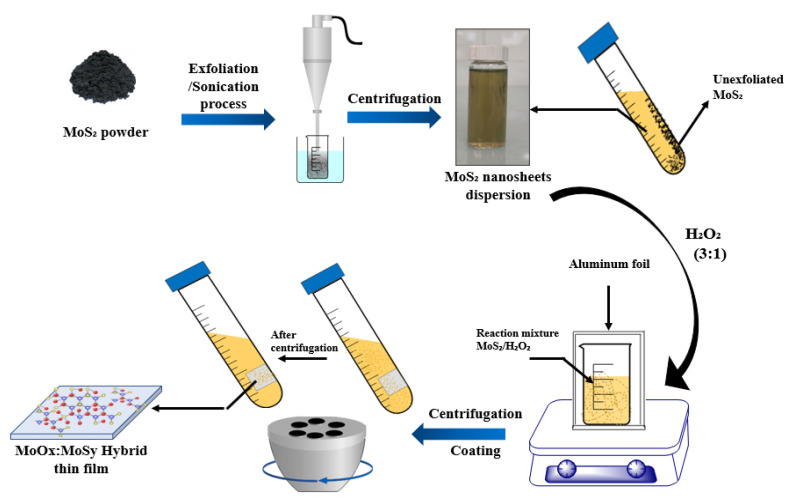
Schematic illustration of MoS_2_ exfoliation and hybrid thin film preparation by centrifugation-coating.

Thin films preparation: Growth of the MoO_3_:MoS_2_ and MoS_2_ thin films was achieved using centrifugal force as a coating technique; this technique is reported in the reference [25], and the exfoliated MoS_2_ is used as the starting dispersion. The deposition process is described in Figure 1.

The hybrid MoO_3_:MoS_2_ thin films were obtained according to the oxidation of MoS_2_, and all the used parameters were kept as described above, except the starting nanodispersion, where the commercial MoS_2_ nanopowder was replaced by the exfoliated MoS_2_ dispersion intending to obtain homogenous thin films. The exfoliated MoS_2_ centrifuged at 600× *g* was used to keep the same mass concentration, 1 mg mL^−1^, as it is adopted in the first protocol of the partial nanopowder oxidation. Note that all the reactions have been released in the dark condition during the hybrid thin film preparation.

The obtained MoS_2_ dispersions after exfoliation/oxidation and the exfoliated MoS_2_ were transferred to another centrifuge tube with graduation of 50 mL where an ITO-coated PET flexible substrate was introduced and stuck on the edge bottom of the centrifuge tube using an adhesive tape. Due to the centrifugal force applied to the dispersion, thin films were deposited uniformly on the flexible substrates that have a dimension of 2.5 × 2.5 cm^2^ or 1 × 2.5 cm^2^. All the thin films were deposited at the relative centrifugal force of 8000 for 10 min using a Sigma 2–16 p centrifuge. With no annealing, the obtained thin films, MoO_3_:MoS_2_ and MoS_2_ layers, were stored in the primary vacuum under pressure of 2.10^−2^ mbar.

The details of the characterization techniques used in this work are given in Appendix A.

## 3. Results

The hybrid MoO_3_:MoS_2_ nanomaterial was synthesized through partial oxidation of MoS_2_ using H_2_O_2_ as an oxidizer. The XPS analyses were performed to check and identify the samples chemical composition after the oxidation process.

The binding energies of the main peaks detected in the XPS spectra and their corresponding components, MoS_2_, MoO_3_, and MoOxSy, extracted from the literature are listed in Table 1.

**Figure 2:**
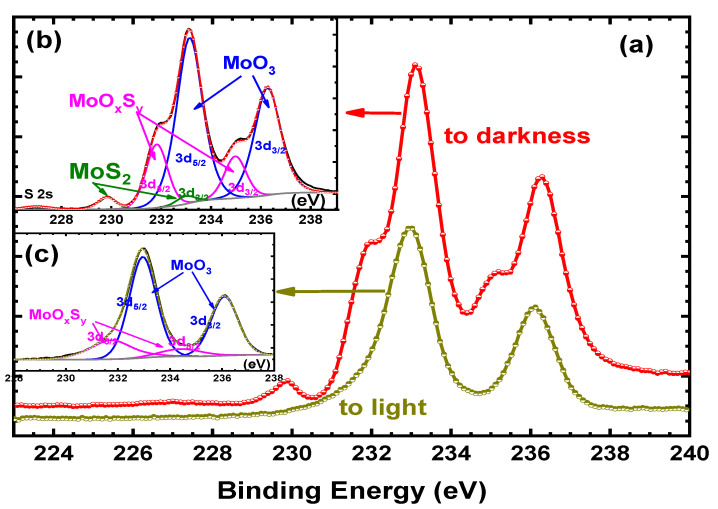
Mo3d spectra of MoS_2_ oxidized by H_2_O_2_ (10%) for 15 min to light and to darkness (**a**). Inset (**b**) Mo3d decomposition after oxidation to darkness and inset. (**c**) Mo3d decomposition after oxidation to light.

First, MoS_2_ was oxidized at room conditions by H_2_O_2_, whose concentration was 10%, for different durations, 30–15 min. Unfortunately, whatever the reaction duration, as shown in Figure 2a,c, no more MoS_2_ was detected. Actually, the Mo 3d spectrum corresponds to two doublets; the first one, situated at 232.9 and 236.0 eV can be attributed to the Mo3d_5/2_ and Mo3d_3/2_ of MoO_3_, while the second one, at 231.7 and 234.8 eV corresponds to MoOxSy (see Table 1) [26]. From these results, MoS_2_ is nearly completely converted to MoO_3_, suggesting full oxidation of MoS_2,_ and even if these results indicate that MoS_2_ is oxidized in the appropriate path to MoO_3_, it does not allow us to achieve one of the main objectives of our work, which is obtaining the hybrid MoO_3_:MoS_2_. Therefore, to try to slow down the oxidation reaction, the samples were put to darkness during the reaction. As visible in Figure 2a,b, for a reaction time of 15 min, some MoS_2_ is still present, indicating that the MoS_2_ oxidation rate decreases when the reaction is carried out in the dark.

**Table 1:** XPS-binding energies of MoS_2_, MoO_3,_ and MoOxSy.

Components		Binding Energies (eV)	References
MoS_2_	Mo 3d _5/2_	229	[23]
	Mo 3d _3/2_	233	
MoO_3_	Mo 3d _5/2_	233	[27,28]
	Mo 3d _3/2_	236	
MoO_x_S_y_	Mo 3d _5/2_	232	[26]
	Mo 3d _3/2_	235	

To be able to control more easily the percentage of MoS_2_ still present at the end of the reaction, we decided to use a small concentration of H_2_O_2_ for the oxidation reaction. Therefore, we proceed to an oxidation using H_2_O_2_ concentrations of 6%. To confirm the influence of light on the oxidation reaction, we compared the Mo3d spectra obtained after 30 min with and without light. The results of the deconvolution of the Mo3d spectra are summarized in Table 2. The atomic percentage of MoS_2_ after 30 min of reaction is nearly double when the reaction is carried out in darkness. Thus, as expected, the MoS_2_ oxidation rate decreases when the synthesis was performed in the dark conditions. Therefore, it is easier to manage the composition of the hybrid material by working in the dark. Therefore, we decided to proceed with oxidation in darkness.

**Figure 3:**
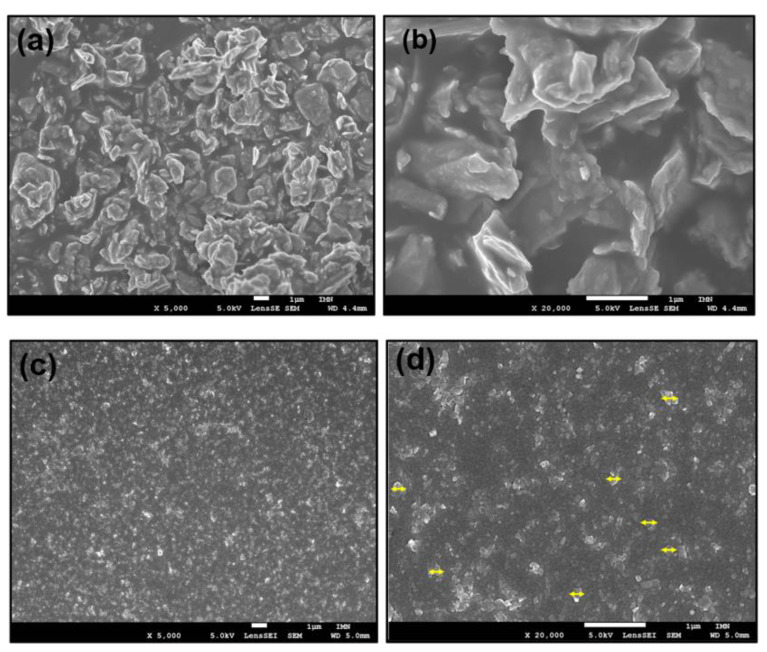
FESEM images of (**a**) MoS_2_ nanopowder and (**b**) MoS_2_ nanpowder at high magnification, (**c**) exfoliated MoS_2,_ (**d**) exfoliated MoS_2_ at high magnification, and the yellow arrows indicate the grain lateral sizes.

The idea was to sweep the composition of the nanopowder from 100% of MoS_2_ not oxidized to 0% after oxidation, using the reaction time as parameter. Unfortunately, when the reaction duration increases beyond 30 min, the oxidation reaction tends to saturate; thus, the longer the reaction duration increases, the faster the oxidation rate decreases. Thereby, while for the 30 min reaction time only 30% of MoS_2_ remains, for 45 min or more, the measured value remains above 20–25%. Indeed, it is an average value because the result changes significantly from one point to another, which suggests that the samples are not homogeneous. If the nominal grain size of MoS_2_ nanopowder is 90 nm, it is in fact an average value, and some grains are far much larger, reaching a size up to 3 µm, as shown in Figure 3a.

The presence or not of such big MoS_2_ grains explains the inhomogeneity of the results and the saturation tendency of the oxidation reaction since it is more difficult to achieve complete reaction in the case of grains of several microns in diameter than in the case of grains of a few tens of nanometers. Since the XPS analysis is essentially a surface analysis, we used the SEM backscattered electron mode [29] to verify the homogeneity of the samples and especially the presence of the MoS_2_ after the oxidation reaction.

**Figure 4:**
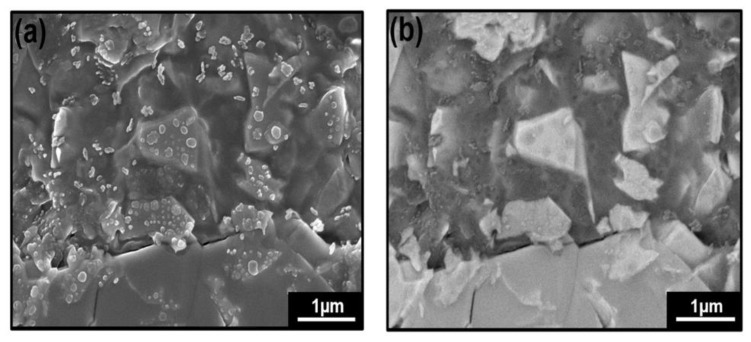
FESEM images of MoS_2_ powder crystal after oxidation of MoS_2_ by H_2_O_2_ 6% for 45 min: (**a**) in the secondary mode and (**b**) in the backscattering mode.

Images of MoS_2_ nanopowder crystal after oxidation by H_2_O_2_ 6% for 45 min in the secondary mode and in the backscattering mode are shown in Figure 4a,b. Lighter areas are visible in Figure 4b; they correspond to non-oxidized MoS_2_ since the heavier atoms backscatter the electrons more than lighter atoms. The dark spots are supposed to correspond to the lightest components, which are the oxidized MoS_2_; thus, these images clearly show the limits of the XPS analysis, as mentioned above. However, a vital point that can be extracted from these results is MoS_2_ does not react homogeneously due to the non-homogenous distribution of MoS_2_ grain size. Starting from the point that H_2_O_2_ can be oxidized and spontaneously exfoliate MoS_2_, as given in previous work [30], it is not beneficial and not easy to directly prepare a homogenous hybrid from MoS_2_ nanopowder; thus, the synthetic process requires another step to keep the control on the reproducibility of the results regarding the critical dependence between oxidation reaction rate and nanoparticles size.

It can be concluded from this study of that it is possible to convert MoS_2_ into MoO_3_ by H_2_O_2_ oxidation, but in addition, we showed that to be able to better manage the reaction rate it was desirable to carry out the oxidation in darkness. Nevertheless, the reaction tends to saturate even when the reaction time increases due to the samples non-homogeneity, as shown by SEM analyses. Therefore, for a controllable oxidation rate, it was necessary to work with a MoS_2_ nanopowder more homogeneous and of real nanometric dimensions. For that reason, we exfoliated MoS_2_ to obtain smaller particles exhibiting more homogeneous dimensions.

Keeping the same preparation strategy of the hybrid MoO_3_:MoS_2_, the liquid phase exfoliation (LPE) was first carried out for MoS_2_ initial powder. LPE, as one of the top-down approaches, is a way to control the 2D MoS_2_ flake morphology and proceed our hybrid synthetic strategy based on MoS_2_ oxidation. Based on recent studies, we believe that the LPE free organic solvent may be a promising technique for specific applications; thus, we proceed in our work for an exfoliation of MoS_2_ in water [17,31,32]. The details of the exfoliation procedure are described in the experimental section.

**Figure 5:**
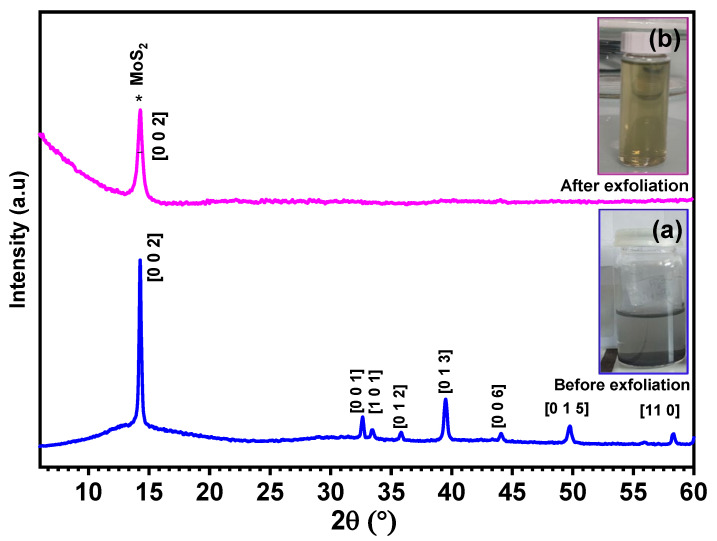
X-ray diffraction of MoS_2_ commercial nanopowder (**a**) and after exfoliation (**b**). The inset figures represent dispersions (**a**) before and (**b**) after exfoliation.

From the photographs showed in Figure 5, the change of the dispersion color from dark gray to yellow color after exfoliation indicate the exfoliation of MoS_2_. To confirm the exfoliation and identify the crystallinity of exfoliated MoS_2_ thin film, the X-ray diffraction technique was conducted on commercial MoS_2_ and exfoliated MoS_2_ samples. Based on the XRD patterns presented in Figure 5a,b, exfoliated MoS_2_ shows a peak with high intensity at 2Ɵ ~ 14° corresponding to the (002) plane of MoS_2_ (PDF.04-004-1905), suggesting that the hexagonal structure of MoS_2_ is retained after exfoliation [17]. The widening of the peak (002) and disappearance of the rest of the peaks after exfoliation compared to MoS_2_ nanopowder indicates the efficient exfoliation of MoS_2_ [33,34].

Before going through the oxidation reaction of the exfoliated MoS_2_, we checked first the sample morphologies using SEM analysis. From the images shown in Figure 3b, we can clearly observe that the particles size was reduced from several microns for commercial powder to 200–250 nm in lateral size at the maximum and with thin flakes for exfoliated MoS_2_. Thus, it confirms the exfoliation of the MoS_2_ nanopowder accompanied the flakes fragmentation during the exfoliation process. As well, we can see the homogenous distribution of the nanoparticles for the sample after exfoliation which is another decisive and important point for our hybrid preparation procedure.

After exfoliation, the nanoparticles are much smaller and more homogeneous than those of the nanopowder; thus, to avoid too rapid oxidation, we used 3% concentration of H_2_O_2_ to achieve the reaction. The oxidation reaction of MoS_2_ and the synthetic route of the hybrid MoO_3_:MoS_2_ thin layer is illustrated in the above scheme (Figure 1).

First, we looked at the reproducibility of the results. To do this, we chose a reaction time of 10 min and a concentration of H_2_O_2_ of 3%. As shown by the atomic percentage of MoS_2_ in Table 3, the results obtained with the exfoliated MoS_2_ are far more reproducible than those obtained with MoS_2_ nanopowder. Changes in the relative concentrations of MoO_3_ and MoOxSy are because the energies of the corresponding doublets are close, which induces a certain margin of uncertainty when the corresponding signal is decomposed.

**Figure 6:**
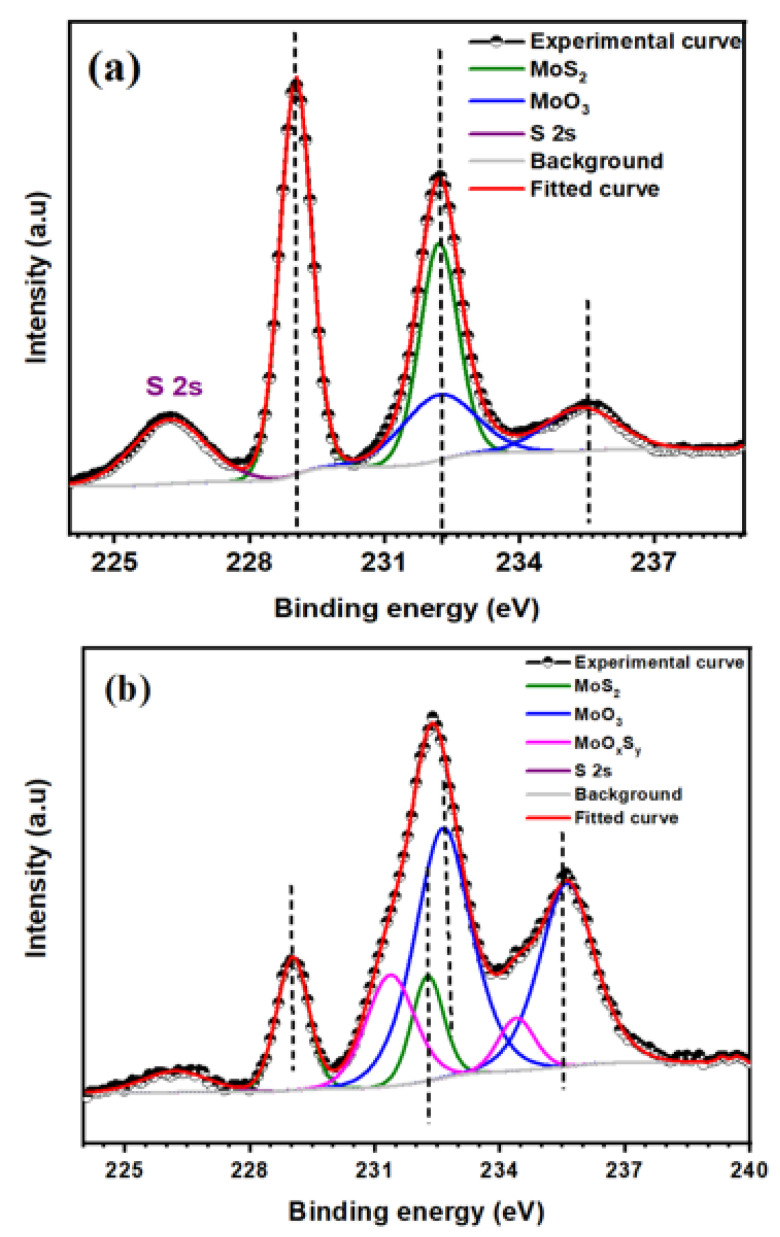
Mo 3d XPS spectra of (**a**) MoS_2_ thin layer and (**b**) MoO_3_:MoS_2_ reaction duration of 10 min.

To better understand the reaction process, we looked at the XPS spectra of all components of the hybrid layer. Firstly, Mo 3d spectra of MoO_3_:MoS_2_ and MoS_2_ thin films deposited on the ITO/PET substrate are presented in Figure 6. From the Mo3d spectrum of MoS_2_ thin film, we can observe two doublets, the first at almost 229 eV and 232 eV corresponding to MoS_2_ and the second doublet at almost 232 eV and 235 eV which correspond to the Mo^5+^ oxidation state that can be attributed to the MoOxSy (Table 1); the presence of a low quantity of this intermediate product in MoS_2_ thin film is coming from MoS_2_ oxidation during the exfoliation process [35]. After MoS_2_ oxidation using H_2_O_2_, we can observe a doublet peak with high intensity at 232.6 eV and 235.6 eV corresponding to MoO_3_ [27] and MoS_2_ at binding energies of 229 eV and 232 eV. The conversion of MoS_2_ to MoO_3_ is accompanied by the third compound at energies of 232 eV and 235 eV corresponding to MoOxSy.

**Figure 7:**
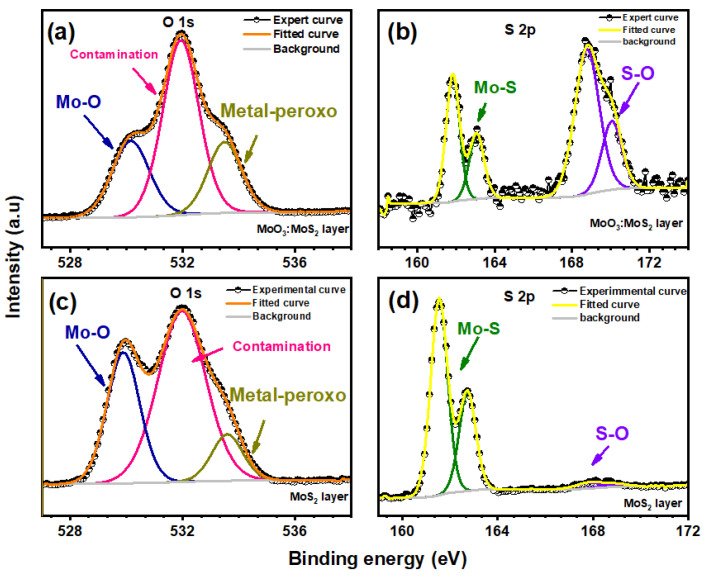
S2p and O 1s zone spectra of MoS_2_ after reaction duration of 10 min (**a**,**b**) and MoS_2_ before reaction (**c**,**d**).

The reaction mechanism of MoS_2_ oxidation is generally described through the reaction of a free hydroxyl radical coming from H_2_O_2_, involved in Mo-S breaking and oxidation of S^2−^ and Mo^4+^ leading to the formation of MoO_3_ [10,36]. However, the oxidation of Mo^4+^ may not be totally achieved since the decomposition of the Mo3d peaks reveals the presence of an intermediate product in the hybrid layer that corresponds to the oxidation state of Mo^5+^; as reported by S. Shin et al., this intermediate corresponds to MoOxSy named molybdenum oxysulfide, which is related to the incomplete substitution of sulfur by oxygen [37,38]. To discuss the reaction process, S 2p and O1s spectra of the hybrid thin film are illustrated in Figure 7; it can be seen in the S2p spectrum that there is a spin-orbit doublet at the binding energy of 161 eV and 163 eV corresponding to the oxidation state S^2−^ which also confirms the existence of MoS_2_ moieties in the hybrid thin film [26]. Moreover, we have detected an additional weak peak at binding energy around 169 eV for the MoS_2_ layer before oxidation [26,38]; the presence of this peak at higher binding energy is mostly attributed to the presence of sulfate (SO_4_^2−^) due to air contamination and the exfoliation process of MoS_2_ as reported in the previous works [35,39]. This peak becomes dominant at almost the same binding energy for the hybrid layer with the appearance of the Mo^5+^ oxidation state which is attributed to the existence of MoOxSy. This finding was also reported by S. Ho Song et al. in their work, where the presence of MoOxSy is accompanied with the appearance of the sulfur peak at higher binding energy of almost 169 eV [26]. Based on the Mo 3d and S 2p spectra, the presence of MoOxSy is related to the association of sulfur and molybdenum with oxygen, which means that the oxygen environment in the hybrid layer is composed of Mo and sulfur. Thus, the presence of Mo-O-S bonds showed after the peak’s deconvolution, giving the new intermediate product the name of oxo-bridge molybdenyl sulfate rather than O-Mo-S bonds. For further details, the deconvolution of O 1 s peaks presented in Figure 7a,c showed the presence of three types of bonding for both oxidized and not oxidized MoS_2_; the peak situated at almost 530 eV is attributed to metal-oxygen (Mo-O), the peak at an energy >531 eV is mostly due to oxygen-sulfur (O-S), and the middle peak detected at an energy of 531 eV which is indicated by “contamination” is attributed to adsorbed contamination and oxygen-carbon that is present in the PET substrate [37]. The peak area of oxygen-sulfur indicates that the amount of O-S bonds increases after MoS_2_ oxidation, and the S2p peak at higher binding energy becomes dominant; this confirms the presence of a small amount of free sulfur oxide accompanied with the new composite oxo-bridge molybdenyl sulfate MoOxSy. A hypothetical structure of this new component produced during the process is illustrated in Figure 9c.

**Figure 8:**
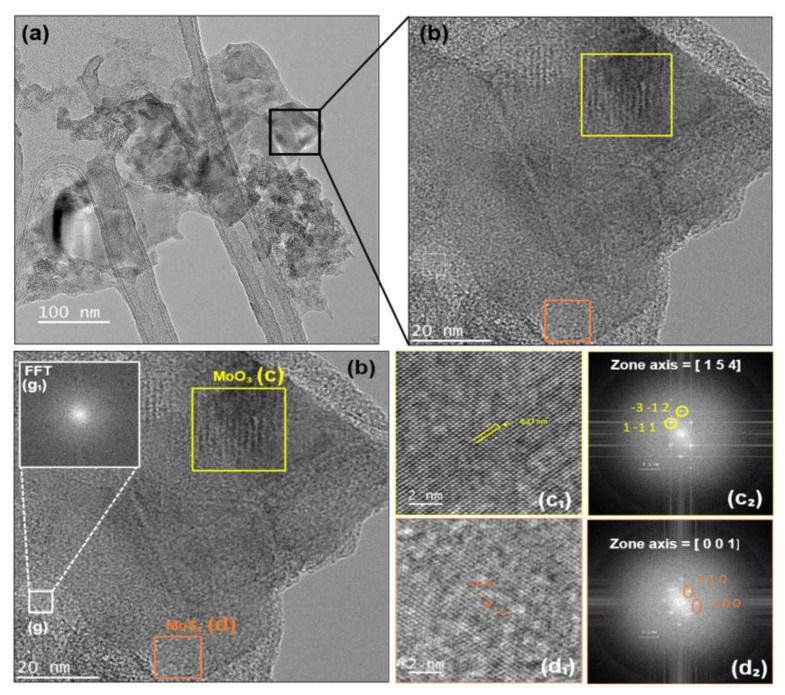
(**a**) HRTEM of MoO_3_:MoS_2_ hybrid nanosheet; (**b**) high magnification image of MoO_3_:MoS_2_ hybrid nanosheet. The (**c**) and (**d**) selected areas with their corresponding high magnification (**c_1_**,**d_1_**) and FFT (**c_2_**,**d_2_**) images indexed with JEMS software [42], respectively; (**g_1_**) FFT image of the selected area (**g**).

To determine the crystallinity of the MoO_3_:MoS_2_ hybrid layer, we did the XRD characterizations; the XRD patterns (not shown) present a weak peak at 14° corresponding to (002) peak of hexagonal structure of MoS_2_ for both the MoS_2_ layer and MoO_3_:MoS_2_ hybrid layer with the presence of peaks derived from the ITO/PET substrate. Thus, this confirms the crystalline structure of MoS_2_ present in the hybrid layer. The absence of additional peaks corresponding to MoO_3_ or the intermediate product, MoOxSy, may be due to the amorphous structure of those compounds or an amount below the detection limit. Furthermore, high-resolution TEM was used to verify the crystalline structure of the studied samples. Figure 8 shows the HRTEM image of the MoO_3_:MoS_2_ hybrid obtained after MoS_2_ oxidation with 30 min of reaction duration; from this image we can observes different zones with a highly crystalline structure as indicated by squares on the image (Figure 8c,d) and other areas with a disordered structure as we can see in Figure 8g. Using fast Fourier transform (FFT) patterns of the selected areas, b_2_, c_2_, and g_1_, we can confirm that the slightly dark color contrast TEM (Figure 8c) is attributed to the crystalline structure of MoO_3_ with a lattice spacing of 0.27 nm, which corresponds to the plan (1–11). The selected area with the orange square (d) reveals the presence of a hexagonal structure of MoS_2_ with a lattice parameter of 0.31 nm [40,41]. As evident in the FFT pattern (Figure 8g_1_), the disordered areas indicate the presence of an amorphous structure which maybe correspond to the oxo-bridge molybdenyl sulfate, MoOxSy, as already revealed by XPS measurements. Briefly, through the oxidation reaction, we obtained nanosheets consisting of a heterostructure containing the three components, MoO_3_, MoS_2_, and MoOxSy.

**Figure 9:**
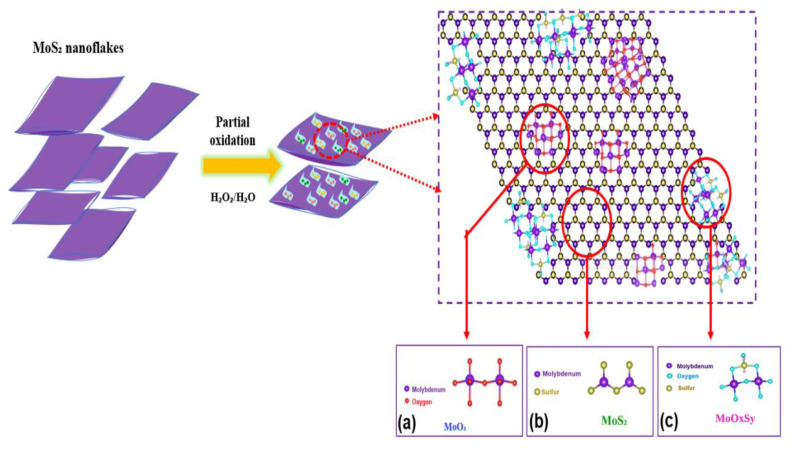
Illustration of the hybrid MoO_3_:MoS_2_ synthetic process, and the chemicals structure of (**a**) MoO_3_ (**b**) MoS_2_ and (**c**) MoOxSy.

Based on the above results, the synthetic process of the hybrid MoO_3_:MoS_2_ produced during the MoS_2_ nanoflakes oxidation can be illustrated as follow (Figure 9).

**Figure 10:**
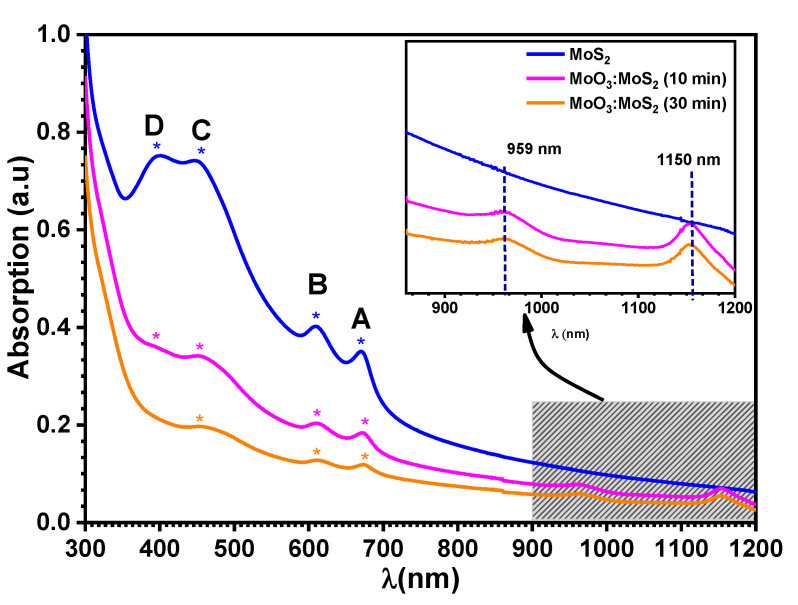
Absorbance spectra of exfoliated MoS_2_ (blue line) and MoO_3_:MoS_2_ dispersion as function of reaction durations of 10 min (pink line) 30 min (orange line).

The absorbance spectra of exfoliated MoS_2_ and the hybrid after reaction durations of 10 min and 30 min are shown in Figure 10. As it is expected, MoS_2_ exhibited four absorption peaks; these peaks are attributed to the excitonic transitions, A, B, C, and D (see Table 4) [43]. From the absorption spectra the estimated bandgap is 1.77 eV for exfoliated MoS_2_. It is known that the thickness reduction shifts the band gap from indirect to direct transition; therefore, herein the increase in the bandgap to 1.77 eV indicates the exfoliation of MoS_2_ to monolayers [17]. Whereas, after the oxidation reaction, the excitonic transitions A and B are almost invariable, which means that the MoS_2_ unreacted during the reaction remains highly crystalline, which is characterized by good absorption in the visible region. Furthermore, in the NIR the hybrid shows two absorption bands at wavelengths of 959 nm and 1150 nm, corresponding to the energies of 1.29 eV and 1.07 eV (inset Figure 10). However, the absorption peaks become weaker when the reaction duration increases due to the decrease in the density of oxygen vacancies [44].

Considering these results, we have studied the evolution of the composition of the hybrid with the time reaction at fixed H_2_O_2_ concentration of 3%. The XPS results are summarized in Table 5.

It can be seen in Table 5 that with the reaction with a H_2_O_2_ concentration of 3% it is possible to manage the atomic concentration of MoS_2_ present in the hybrid layer from 25% after 5 min of reaction to 2.5% after 45 min. It is thus possible to obtain the desired composition for the hybrid layer according to its intended use.

From Figure 6 and Table 5, after 10 min of reaction duration, 60% of MoO_3_ was produced from the MoS_2_ oxidation process after 10 min and 66% after 30 min, giving the MoO_3_/MoS_2_ ratios of 3.5 and 16, respectively. Hence, the MoO_3_:MoS_2_ ratios increase with the time reaction suggesting that there is a relation between the atomic percentage and the reaction duration, where the MoS_2_ atomic percentage decreases and MoO_3_ increases by increasing the reaction duration. The new component MoO_x_S_y_ atomic percentage increases with the reaction duration until its saturation at 15 min with a maximum percentage of 43%, and then the atomic percentage starts to decrease with the reaction duration; this indicates that the MoO_x_S_y_ content also depends on the reaction and the content of oxygen incorporated. Regarding these results, the control of the reaction duration allows us to obtain a high MoO_3_ atomic percentage, which is an essential advantage for the use of the hybrid MoO_3_:MoS_2_ in optoelectronic application and especially in OPVs as the hole transport layer (HTL). The operation of the HTL hybrid layer depends on the atomic percentage of MoO_3_ which must be dominant and that is confirmed by our previous work. We note that, the synthesis of the hybrid MoO_3_:MoS_2_ layer in our previous work was carried out using sulfidation of MoO_3_ thin film, and the control of its composition was achieved by varying the annealing temperature of the substrate. The application of this hybrid as the hole transport layer in the planar heterojunction solar cells gives better results when the atomic percentage of MoS_2_ in the hybrid layer does not exceed 5 %, which corresponds to 65% of the MoO_3_ atomic percentage; therefore, the presence of a high MoS_2_ content (>5%), which means low MoO_3_ concentrations in the hybrid MoO_3_:MoS_2,_ could limit the positive effect of the hybrid layer when used as HTL. Taking into account these previously obtained results, the presence of such a high MoO_3_ percentage of about 65% in the MoO_3_:MoS_2_ layer as HTL is beneficial for enhancing the photovoltaic performances due to the improvement of the band matching at the electrode and photoactive layer interface [24]. On the basis of this finding, we can conclude that controlling the atomic percentage of such a component of the hybrid layer is necessary to achieve good device performances when the hybrid is introduced; thus, the successful control of the composition achieved in the present work could open up the use of the hybrid prepared by wet chemical synthesis as the hole transport layer (HTL) in the organic solar cells.

Herein, the measurements of the bandgap from the absorption spectra are uncertain; hence, the bandgap is stable at 1.77 eV ± 0.02 for MoS_2_ and oxidized MoS_2_. However, the presence of defects under the bandgap due to the presence of the oxo-bridge molybdenyl sulfate, MoO_x_S_y_, can improves the holes extractions when it is applied as HTL in solar cells [8]. In a previous report, the MoS_2_ bandgap can be tuned after its oxidation depending on the MoO_x_S_y_ atomic percentage where they find that the bandgap extended into the visible range when the MoO_x_S_y_ contents increased. The MoO_x_S_y_ reaching 50 at.% induces an increase of the bandgap by 0.25 eV, proving that the modulation of the bandgap is possible by controlling the MoO_x_S_y_ contents [26]. Thus, this indicates that the presence of the MoO_x_S_y_ composite can be efficient to reach a wide bandgap energy. In addition, the presence of these defects in the hybrid layer can effectively enhance the absorption ability of MoS_2_ towards polysulfides, as reported for Li-S batteries [10].

## 4. Conclusions

In conclusion, we have reported a facile synthetic route to prepare the hybrid MoO_3_:MoS_2_ layer via exfoliation/partial oxidation of MoS_2_ under ambient temperature. Before investigation of the properties of the hybrid layer, the first part was detected in the partial oxidation of MoS_2_ nanopowder where we found that the reaction can be controlled efficiently in the darkness, and the homogeneity of the grain sizes is needed to obtain high reproducibility of the results. To overcome the non-homogenous results, the second part was dedicated to the exfoliation by LPE to control the grain size homogeneity and therefore, the morphological, optical, and structural properties of the MoS_2_ and the hybrid MoO_3_:MoS_2_ layer.

In the present study, an original method of LPE was done in pure water as the solvent; the use of water is beneficial as it is not only cost-effective in industrial and commercial applications but also facilitates the hybrid synthesis via the MoS_2_ oxidation. A possible growth mechanism of MoS_2_, the MoO_3_:MoS_2_ thin layer was achieved using centrifugation/coating method.

The Investigation of XPS analyses revealed the presence of the three compounds, MoO_3_, MoS_2,_ and MoOxSy; this heterostructure was also confirmed by HRTEM analyses. Based on the evolution of the reaction, we found that the atomic percentage of each component depends on the reaction duration; the MoO_3_ atomic percentage can exceed 60%, and the presence of the oxo-bridge molybdenyl sulfate, MoO_x_S_y_, in the hybrid layer justifies the possibility to use this hybrid for large potential applications, especially solar cells, Li-batteries, and catalysis.

## Figures and Tables

**Table 2:** Composition of the hybrid nanopowder after oxidation of MoS_2_ by H_2_O_2_ 6% for 30 min, in function of light environment: to light or in the dark.

Experimental Conditions	MoS_2_ (at%)	MoO_3_ (at%)	MoOxSy (at%)
To light	17	71	12
To darkness	30	60	10

**Table 3:** Atomic percentages extracted from Mo 3d XPS spectra of the components present in the MoO_3_:MoS_2_ hybrid thin film after 10 min of reaction in the darkness, the H_2_O_2_ concentration being 3%.

Sample	MoS_2_ (at%)	MoO_3_ (at%)	MoOxSy (at%)
N°1	18	62	20
N°2	17	56	27
N°3	16	66	17

**Table 4:** Positions of the excitonic peaks.

Excitonic Transition	Exfoliated MoS_2_	Hybrid MoO_3_:MoS_2_	
		10 min	30 min
A	670	673	673
B	607	610	610
C	448	454	454
D	398	398	-----

**Table 5:** Evolution with the reaction time of the composition of the hybrid thin film (3% H_2_O_2_, in the darkness), based on decomposition of Mo 3d peaks.

Reaction Duration (min)	MoS_2_ (at%)	MoO_3_ (at%)	MoOxSy (at%)
5	25	56	19
10	17	60	23
15	5	52	43
30	4	66	30
45	2.5	75	23

## Data Availability

Not available.

## Appendix A

Chemical compositions of the synthesized hybrid MoO_3_:MoS_2_ nanopowder and thin films were investigated by X-ray photoelectron spectroscopy (XPS) analysis at room temperature using the Axis Nova spectrometer (Kratos Analytical) and employing Al Kα line as the excitation source. The spectra were adjusted according to the fixed binding energy of carbon C 1 s at 284.8 eV as a reference. The XPS data were treated using CasaXPS software, the fitting was obtained by a mixed Gaussian–Lorentzian product with 30% and 70% as the contribution of Lorentzian and Gaussian, respectively, for Mo 3d fitting the area ratio of 2:3 was fixed for Mo 3d 5/2 and Mo 3d 3/2, respectively, after the extraction of the Shirley background. The core-level spectra were recorded using a constant pass energy of 20 eV with an energy step of 0.1 eV.

The morphology of the studied hybrid nanomaterials was analyzed using a scanning electron microscopy (SEM) with a JEOL JSM 7600F field-emission scanning electron gun microscope (Centre de micro-caractérisation, Institut des Matériaux de Nantes Jean Rouxel IMN, Nantes université). Images were recorded using a secondary electron detector and a backscattered electron detector, and the accelerating voltage was set to 5 KV. Transmission electron microscopy (TEM) and high-resolution TEM (HRTEM) images were collected by a S/TEM Themis Z G3 (thermo Fisher Scientific) microscope with an operating voltage at 300 KV. The samples analyzed by TEM were prepared by the deposition of a droplet on Cu lacey carbon grid.

Optical absorption spectra were measured at room temperature using a UV-visible-NIR spectrophotometer (Perkin Elmer Lambda 1050 setup) at a normal incidence in the spectral range 300 nm–1200 nm.

X-ray diffraction was done with Diffractometer Burker D8 A25 “Da Vinci” setup using Cu Kα radiation (λ_Kα_ = 1.5418 Å); the diffractometer is equipped with a second-generation Si strip detector (“LynxEye XE”).
